# Impact of chromosomal instability on colorectal cancer progression and outcome

**DOI:** 10.1186/1471-2407-14-121

**Published:** 2014-02-22

**Authors:** Béatrice Orsetti, Janick Selves, Caroline Bascoul-Mollevi, Laurence Lasorsa, Karine Gordien, Frédéric Bibeau, Blandine Massemin, François Paraf, Isabelle Soubeyran, Isabelle Hostein, Valérie Dapremont, Rosine Guimbaud, Christophe Cazaux, Michel Longy, Charles Theillet

**Affiliations:** 1 INSERM U896, F-34298 Montpellier, France; 2 Institut de Recherche en Cancérologie de Montpellier, Université Montpellier1, F-34298 Montpellier, France; 3 Institut régional du Cancer Montpellier, F-34298 Montpellier, France; 4 University Hospital of Purpan, Toulouse, France; 5 Cancer Research Center of Toulouse – UMR 1037 INSERM, University of Toulouse, Toulouse, France; 6 Centre Hospitalier Universitaire Dupuytren and EA 3842 Faculté de Médecine, Limoges, France; 7 Cancer genetics unit, Institut Bergonié, Bordeaux, France; 8 University Hospital of Rangueil, Toulouse, France; 9 Cancer Research Center of Toulouse, INSERM U1037 CNRS ERL5994, University Paul Sabatier, University of Toulouse, Toulouse, France; 10 INSERM U916, Institut Bergonié, Université de Bordeaux, Bordeaux, France; 11 Institut de Recherche en Cancérologie de Montpellier, INSERM U896, 208 Rue des apothicaires, 34298 Montpellier cedex 5, France

**Keywords:** Colorectal cancer, Genomic instability, Breakpoint, Array CGH, CIN tumors, Adenoma, Primary tumors, Metastasis, Outcome, 16p13.3, 19q13.3

## Abstract

**Background:**

It remains presently unclear whether disease progression in colorectal carcinoma (CRC), from early, to invasive and metastatic forms, is associated to a gradual increase in genetic instability and to a scheme of sequentially occurring Copy Number Alterations (CNAs).

**Methods:**

In this work we set to determine the existence of such links between CRC progression and genetic instability and searched for associations with patient outcome. To this aim we analyzed a set of 162 Chromosomal Instable (CIN) CRCs comprising 131 primary carcinomas evenly distributed through stage 1 to 4, 31 metastases and 14 adenomas by array-CGH. CNA profiles were established according to disease stage and compared. We, also, asked whether the level of genomic instability was correlated to disease outcome in stage 2 and 3 CRCs. Two metrics of chromosomal instability were used; (i) Global Genomic Index (GGI), corresponding to the fraction of the genome involved in CNA, (ii) number of breakpoints (nbBP).

**Results:**

Stage 1, 2, 3 and 4 tumors did not differ significantly at the level of their CNA profiles precluding the conventional definition of a progression scheme based on increasing levels of genetic instability. Combining GGI and nbBP,we classified genomic profiles into 5 groups presenting distinct patterns of chromosomal instability and defined two risk classes of tumors, showing strong differences in outcome and hazard risk (RFS: p = 0.012, HR = 3; OS: p < 0.001, HR = 9.7). While tumors of the high risk group were characterized by frequent fractional CNAs, low risk tumors presented predominantly whole chromosomal arm CNAs. Searching for CNAs correlating with negative outcome we found that losses at 16p13.3 and 19q13.3 observed in 10% (7/72) of stage 2–3 tumors showed strong association with early relapse (p < 0.001) and death (p < 0.007, p < 0.016). Both events showed frequent co-occurrence (p < 1x10-8) and could, therefore, mark for stage 2–3 CRC susceptible to negative outcome.

**Conclusions:**

Our data show that CRC disease progression from stage 1 to stage 4 is not paralleled by increased levels of genetic instability. However, they suggest that stage 2–3 CRC with elevated genetic instability and particularly profiles with fractional CNA represent a subset of aggressive tumors.

## Background

Genetic instability is a hallmark of cancer cells and has been proposed to act as a catalyst of cancer development from early stages on [1,2]. It is generally agreed that tumor progression occurs according to a scheme of gradual accumulation of genetic anomalies and that genetic instability is highest in most aggressive and metastatic forms of the disease. In colorectal cancer (CRC), genetic instability is subdivided into three classes; (i) mismatch repair deficiency (MIN), often of hereditary origin but also sporadically acquired, associated with base slippage mostly at poly(A) or poly(C) tracks and near diploid genomes, 15% of CRC (ii) chromosomal instability (CIN) resulting in severely rearranged karyotypes and aneuploidy, 65% of CRC (iii) non-MIN/non-CIN showing a methylator phenotype, 20% of CRC [3]. Major genetic mutations found and acting as key events in CRC, affect the *WNT/APC/CTNNB1*, *KRAS/BRAF*, *FBXW7*, *PTEN*, *SMAD4*, *TGFBRII*, and *TP53* genes [4,5]. Interestingly, patterns of mutated genes vary according to the class of CRC. *BRAF* mutations seem prevalent in MIN, whereas *TP53* mutations are essentially found in CIN. Interestingly, genes promoting DNA repair, DNA damage checkpoint as well as translesional DNA replication are mostly down-regulated in CRC tumors compared to proliferating normal adjacent tissues, probably favoring the overall genetic instability at the nucleotide level [6]. In addition to these functionally validated aberrations, CGH based studies have identified widespread copy number alterations (CNA), some of which highly recurrent. Typical CNA patterns in CRC involve gains at 8q, 13q and 20q as well as losses at 5q, 8p, 17p and 18q [7]. These observations were confirmed in higher resolution array-CGH analyses and the boundaries of these regions of CNA defined with greater precision. Moreover, a number of focal events were pointed out [8]. The number of genetic anomalies linked to CRC pathogenesis is elevated and has risen with recent large scale sequencing efforts [9]. However, questions remain as to the role of widespread chromosomal instability in the course of the disease, in particular how these relate to progression of CRCs and patient relapse.

Although the sequential order originally proposed by Fearon and Vogelstein for CRC progression has been disputed the overall model is still regarded as valid [10]. Stepwise progression from normal epithelium, through dysplasia to carcinoma builds on a gradual accumulation of genetic anomalies. Recent work showed that copy number alterations (CNA) set in early in adenomas and reached in progressed adenomas a level similar to that found in carcinomas [6,11]. It has also been suggested on the basis of a meta-analysis of chromosome CGH [7] and array-CGH [8] that progression from invasive cancer to metastasis was accompanied by an increase in the number of CNAs.

However, no clear cut results were proposed ascertaining the existence of a molecular progression scheme between early carcinoma (stage 1), invasive (stage 2 and 3) and metastatic (stage 4) CRC. In this work we wanted to verify whether we could relate CRC progression (from stage 1 to stage 4 and, eventually, to distal metastasis) to a gradual increase of genetic instability and sketch out a sequence of CNA increment. Moreover, we wanted to determine whether genetic instability correlated with patient outcome. To this aim we analyzed a set of 162 CIN CRCs comprising 131 primary carcinoma evenly distributed through stage 1 to 4 and 31 metastases (28/31 formed a primary-tumor/matched-metastasis pair) and 14 adenomas by array-CGH. Our data showed that stage 1, 2, 3 and 4 tumors did not differ significantly at the level of their CNA profiles. This led us to ask whether the level of genomic instability, as illustrated by array-CGH, was linked to disease outcome. Based on the Global Genomic Index (GGI), which corresponds to the fraction of the genome involved in CNA and the number of breakpoints (nbBP), which were determined as chromosomal sites where copy number shifts occurred, we defined two classes of tumors showing strong differences in outcome and hazard risk. CNAs correlating with early relapse or death in stage 2 or 3 patient were searched and two regions of copy number loss could be selected due to their strong association to negative outcome.

## Methods

### Patient and tumor samples

Genomic profiles were established on 176 samples: 14 adenomas, 131 primary carcinoma and 31 synchronous (9) or metachronous (20) metastases (among which 28 were paired to their primary tumor). Biological samples were collected in 4 clinical centers of south-west France: Bergonié Institute, Bordeaux; CHU Dupuytren, Limoges; CRLC Val d’Aurelle, Montpellier; Purpan Hospital, Toulouse between 1993 and 2008. Clinical data and follow-up information were collected. Data were anonymized. This project was submitted to the ethics committees of the respective clinical centers participating to the study and was approved by the National Institute of Cancer (INCa) following the recommendations of the French National Authority for Health (FNAH). Patient samples were processed according to French Public Health Code (law n°2004-800, articles L. 1243–4 and R. 1243–61) and the four biological resources center has received the agreement from the French authorities to deliver samples for scientific research. The authorization numbers were AC-2008-812 (Bergonié), AC-2007-34 (Dupuytren), AC-2008-700 (Val d’Aurelle), AC-2008-820 (Purpan). Before surgery patients are informed that their surgical specimens can possibly be used for research purposes. They can refuse this possibility by filling a form to express refusal and in this case tumor biopsies were destroyed.

Clinical characteristics are summarized in Table 1 and further detailed in Additional file 1. Adenomas and carcinomas were surgically removed and immediately frozen at - 80°C. Only samples containing more than 50% of tumor cells were included in the study. Samples were checked for microsatellite instability by microsatellite marker analysis and were all MIN negative. Four (4) patients (TNM stage 4) received a treatment prior to surgery.

**Table 1:** Patient and tumor characteristics of adenoma and colorectal cancers included in the study

		**Adenomas**		**Carcinomas-primary tumors**	
		**N=14**	**%**	**N=131**	**%**
**Age (y)**					
	<=60	6	42.9	39	30.2
	>60	8	57.1	90	69.8
	Missing	0		2	
**Gender**					
	Male	9	64.3	75	57.3
	Female	5	35.7	56	42.7
**Localisation**					
	Colon	4	28.6	7	5.4
	Left Colon	5	35.7	47	36.4
	Right Colon	4	28.6	31	24.1
	Rectum	1	7.1	44	34.1
	Missing	0		2	
**Node Status**					
	N0			66	51.2
	N+			63	48.8
	Missing			2	
**Stage TNM**					
	I			20	15.5
	II			45	34.9
	III			27	20.9
	IV			37	28.7
	Missing			2	
**Survival Status**					
	Alive			84	65.1
	Dead			45	34.9
	Missing			2	
**Relapse**					
	No			71	56.8
	Yes			54	43.2
	Missing			6	
**Quantitative Genomic**					
**Variables**	GGI median [range]	0.12 [0.03-0.54]			0.35 [0.04-0.64]
	nbBP median [range]	65.5 [50-99]			76 [33-203]

*Abbreviations*: *TNM* Tumor Node Metastasis staging UICC/AJCC 6^th^ edition, *GGI* Global Genomic Index.

*nbBP* number of breackpoints; *RFS* relapse-free survival, *OS* overall survival.

### DNA extraction

Genomic DNA was extracted using QIAmp DNA mini kit (Qiagen, Courtaboeuf, France). Each DNA sample was quantified by nanospectrophotometry (NanoView, GE Healthcare, Orsay, France) and qualified by 0.8% agarose electrophoresis.

### *TP53* mutation

*TP53* mutation status was determined in 98 samples by sequencing 3 PCR fragments containing exons 5 to 9 (Genoscreen, Lille, France). PCR reactions were done using BDT v3.1 kit in a DNA thermocycler PCR 9700 (Applied Biosystems, Villebon-sur-Yvette, France). Each sample was sequenced on both sense and antisense strands on a 96-capillary 3730xl DNA Analyzer. PCR primers used for amplification were the following: P53_ex5-6-F:TGAGGTGTAGACGCCAACTCT, >P53_ex5-6-R: TAGGGAGGTCAAATAAGCAG, >P53_ex7-F: CCTGCTTGCCACAGGTCT, >P53_ex7-R: TCTACTCCCAACCACCCTTG, >P53_ex8-9-F: CAAGGGTGGTTGGGAGTAGA, >P53_ex8-9-R : TGTCTTTGAGGCATCACTGC.

Mutation detection was then done by sequence alignment and comparison to the Genebank reference sequence NC_000017 (7512445..7531642) using Multalin (http://bioinfo.genotoul.fr/multalin/). Each mutation was validated using the mutation validation tool available on IARC TP53 database (http://www-p53.iarc.fr/).

### Array-CGH

The 176 DNA samples were analyzed on two generations of Integragen BAC-arrays (Integragen, Evry, France) IgV6+ (5015 BACs), IgV7 (5878 BACs), with a median resolution of 0.6 Mb. BACs were spotted in quadruplicate. DNA labeling and hybridization, were done as previously described [12] with slight modifications: 600 ng of DNA were labeled with BioPrime Total Genomic Labeling System (Invitrogen SARL, Cergy Pontoise, France). Arrays were scanned using Axon 4000B scanner (Molecular Devices, CA, USA) and images were analyzed using Genepix 6.0. Data were analyzed in web-based platform for copy number array management and analysis (http://bioinfo-out.curie.fr/CAPweb/). Normalized and replicates filtered data were exported as text file for further analyses. In order to analyze all the data from different Integrachip versions, we used the Nexus 6.0 Software (Biodiscovery, El Segundo, CA, USA). Analysis settings for data segmentation and calling were the following: significant threshold for Rank Segmentation algorithm: 0.005, Max Continuous Probe Spacing: 6000, Min number of probes per segment: 6, high level gain: 0.485, gain, 0.138, loss:-0.153, homozygous copy loss:-0.73. Nexus 6.0 Software was used to calculate frequency plots, factor enrichment (significantly overrepresented factor values in a particular factor group identified using the two tailed Fisher’s Exact test at a p-value of p < 0.05), significant chromosomal differences between two groups (comparison tool: two tailed Fisher’s exact test with p-value < 0.005 and minimal frequency difference set at 10%) and Survival Predictive Power (log-rank test is used to identify genomic regions yielding a high degree of survival prediction; p-value is calculated by permuting the survival time for each sample and comparing the log-rank statistic for the permuted data to the original data; threshold used was p-value < 0.05).

### Genomic quantitative variables calculation

An R script using Circular Binary Segmentation (CBS) algorithm implemented in DNAcopy (Bioconductor for R) and normalized/replicates filtered data as input, were used to determine genomic metrics such as gains, losses, high level gains, homozygous copy losses. For this purpose, the thresholds were as used in Nexus 6.0 analysis (high level gain: 0.485, gain, 0.138, loss:-0.153, homozygous copy loss:-0.73). The GGI was calculated at a probe level as follows: (number of probes gained + number of probes lost) /number of informative probes. The GGI corresponds to the fraction of the genome involved in CNA. The nbBP was determined as the number of transitions or breakpoints in the genomic profiles after smoothing and segmentation of the data. The R script is available upon request.

### Statistical analyses

Continuous variables were presented as medians and range, and compared between populations with the Kruskal-Wallis test. Categorical variables were presented using contingency tables and compared with Pearson’s chi-square test or Fisher’s exact test. Differences were considered statistically significant when p < 0.05. Classes of genetic instability were defined using two quantitative variables as metrics: Global Genomic Index of alteration (GGI) and number of breakpoints (nbBP). First on the whole set of data (n = 176), GGI and nbBP were grouped into three classes using the 33th percentile (first tercile) and the 66th percentiles (second tercile). Then, for stage 2 and 3 set of data (n = 72), number of BP was grouped into two classes, low (<116) and high (>116). Using ROC curves (see Additional file 2) the optimal nbBP threshold was calculated to maximize the Youden’s index (sensitivity and specificity minus 1) which induces the best discrimination according to vital status. Statistical associations between GGI or nbBP were calculated using the nonparametric test for trend across ordered groups. To account for multiple testing, the statistically significant threshold was set at 0.01.

Overall survival (OS) was the primary endpoint for this study and was calculated from the date of surgery until the date of death. Relapse-free survival (RFS) was the secondary endpoint and was calculated from the date of surgery until the date of relapse. Patients who died without relapse were censored at the time of death. Patients lost to follow-up were censored at the time of last visit. The Kaplan-Meier method was used to estimate OS and RFS. Survival rates were compared using log-rank test.

Genomic instability variables were significant in univariate analysis and were included into a multivariate Cox proportional hazards model. Using the model, a score was allocated proportional to the regression coefficients. The adjacent non-significant categories were regrouped in order to reduce the number of prognostic categories (see Additional file 3). Hazard rate (HR) and its 95% confidence interval (95% CI) were calculated using Cox model.

Statistical analyses were performed with GraphPad Prism 5 (http://www.graphpad.com) and STATA software 11.0 (StatCorp. 2009. Stata: Release 11. Statistical Software. College Station, TX: StataCorp LP).

## Results

### Outcomes in our colorectal cancer set

Median follow-up was 48.4 months (range: 1 to 115 months). Median overall survival was not reached. Three-year relapse-free survival (RFS) was 69% (95% CI: 55–79) and 5-years overall survival (OS) was 66% (95% CI: 51–78).

### Copy number alterations in our colorectal cancer set

**Figure 1:**
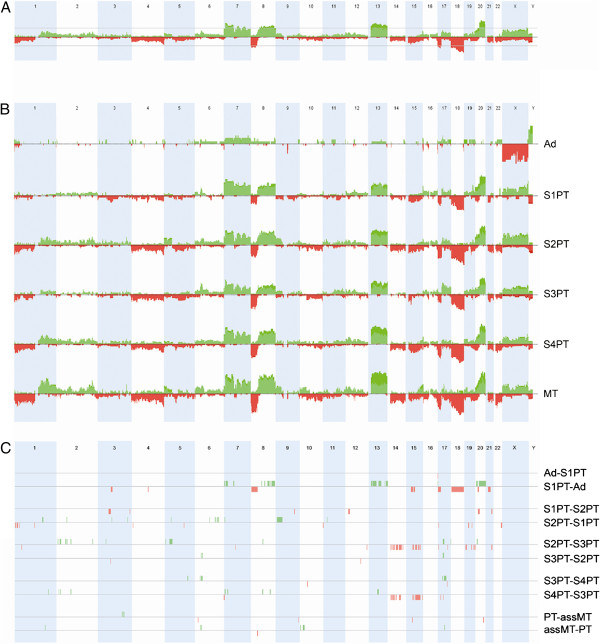
**Copy number alteration (CNA) patterns.** Gains are shown in green and losses in red. Boundaries of chromosomes are indicated by white and blue vertical areas. **A**: CNA frequency plot in the complete tumor set. The grey horizontal bar indicates the 35% of tumors affected threshold. **B**: CNA frequency plots in different disease stages: Ad: adenomas, S1PT: Stage 1 Primary Tumors, S2PT: Stage 2 Primary Tumors, S3PT: Stage 3 Primary Tumors, S4PT: Stage 4 Primary Tumors, MT: metastases. **C**: significant differences between sequential steps of disease progression. assMT: depicts metastases associated to its cognate primary tumor.

Genomic profiles were established on our set of 176 colorectal tumors by CGH on BAC-arrays comprising 3000 to 5800 clones (mean resolution 1 to 0.6 Mb). Our sample set corresponded to 14 adenomas, 131 primary tumors and 31 distal metastases. All these CRC samples were selected as microsatellite stable. Overall CNA profiles in our set of tumors were in harmony with those described by others ([8,13]) (Figure 1A). Most commonly altered regions (gains or losses in > =35% of the samples) were gains at chromosomes 7p, 7q, 8q, 13q, 20 and losses at 8p, 17p and 18 (Figure 1A, Additional file 4). High-level gains (HLG) (log2ratio > 0.485) were observed throughout the whole genome. However, only HLGs located at 7p21.3-p11.2, 8q11-q24.3, 13q11-q34 and 20p13-q13.33 occurred in more than 5% of the tumors.

### Stratification of CNA profiles and genetic instability according to disease stages

We wanted to determine the existence of copy number changes correlated to disease progression from adenomas to carcinomas and from superficial (stage 1) to invasive (stage 2 and 3) and metastatic cancer (stage 4 and metastases). To this aim, we stratified CGH profiles according to disease stages and metastases (Figure 1B). Adenomas clearly differed from carcinomas showing less rearranged profiles. This indicated that the transition from benign to malignant tumors was accompanied by a sharp increase in genetic instability. Contrastingly and interestingly, cumulative CNA profiles of stage 1, 2, 3, 4 carcinomas and distal metastases appeared globally similar. To identify regions of CNA associated to the progression from one disease stage to another, we performed pairwise comparisons (adenomas vs. stage 1 carcinomas, stage 1 vs. stage 2, 2 vs. 3, 3 vs. 4 and metastases vs. associated primary tumors) (Figure 1C and Additional file 5). Most significant changes were seen between adenomas and stage 1 carcinomas, with gains at 8q, 13q, 20 and losses at 8p, 15p, 17p and 18q. Changes associated to stage transition (1 to 2, 2 to 3, 3 to 4) could be found, but were difficult to relate to a coherent scheme of progression. This was exemplified by losses at chromosome 14 and 15, associated to transition from stage 2 to 3 and from stage 3 to 4 (Figure 1C). Both events were present in stage 2 and absent in stage 3. Strangely, their occurrence went back up in stage 4. This was not consistent with a cumulative progression scheme, in which tumors progress sequentially from stage 2 to stage 3 and end up progressing to stage 4.

**Figure 2:**
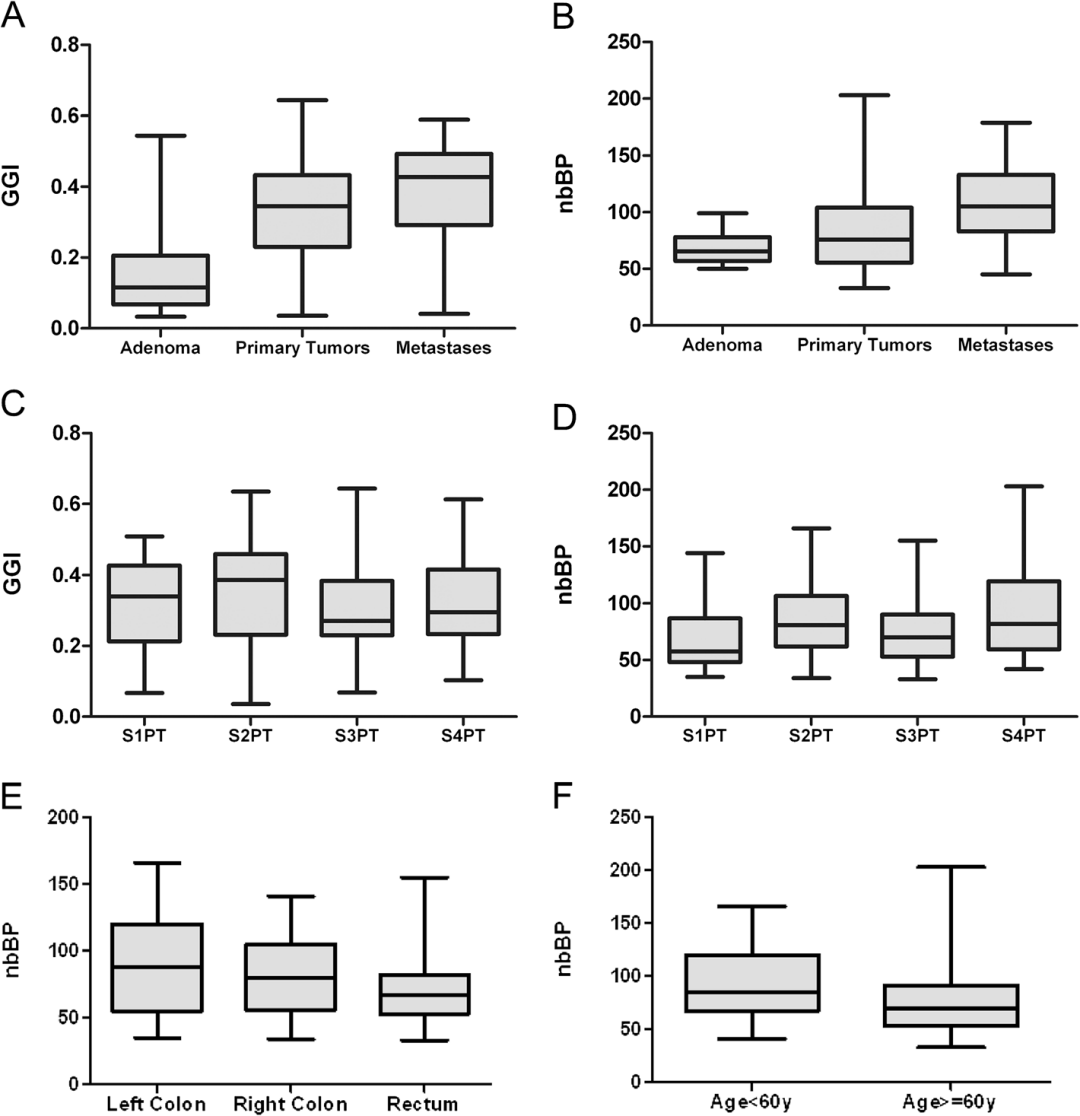
**Distribution of genomic instability as defined by the GGI and nbBP metrics in different groups of colorectal tumors. A**: Boxplots of GGI values in adenoma, primary CRC and metastases. **B**: Boxplots of nbBP values in adenoma, primary CRC and metastases. **C**: Boxplots of GGI values in the 4 stages of CRC: stage 1 (S1PT), stage 2 (S2PT), stage 3 (S3PT), stage 4 (S4PT). **D**: Boxplots of nbBP values in the 4 stages of CRC: stage 1 (S1PT), stage 2 (S2PT), stage 3 (S3PT), stage 4 (S4PT). **E**: Boxplots of nbBP values in different location of colon cancer (left colon, right colon and rectum). **F**: nbBP boxplots stratified on patient age.

Next, we verified whether the level of genetic instability increased according to disease stage using two metrics, Global Genomic Index (GGI), and the number of breakpoints (nbBP) (as defined in the Materials and Methods section). Median levels [range] of GGI and nbBP in the whole dataset were 0.35 [0.03 – 0.64] and 81 [33 – 203] respectively. It was apparent that genetic instability increased significantly between adenomas (AD) (GGI = 0.12/nbBP = 65.5), primary tumors (PT) (GGI = 0.35 / nbBP: 76) and metastases (MT) (GGI: 0.43 / nbBP: 105); (AD vs PT p = 0.0001, PT vs MT p = 0.005) (Figure 2A, B), but did not change significantly from stage 1 to 4 carcinomas (Figure 2C, D). We delineated classes of genetic instability based on GGI or nbBP terciles and were intrigued to see that stage 2 and 4 presented a large proportion of GGI-high and/or nbBP-high tumors, while stage 1 showed a prevalence of low instability tumors (see Additional file 6A, B). Moreover, we noted that the nbBP was higher in younger patients (<60y, p = 0.013) and lower in rectal cancers (p = 0.032), irrespective of TNM stage (Figure 2E-F). However, GGI and nbBP levels did not differ significantly between *TP53* wild type and their mutated counterparts.

### Genetic instability and outcome of the disease

**Figure 3:**
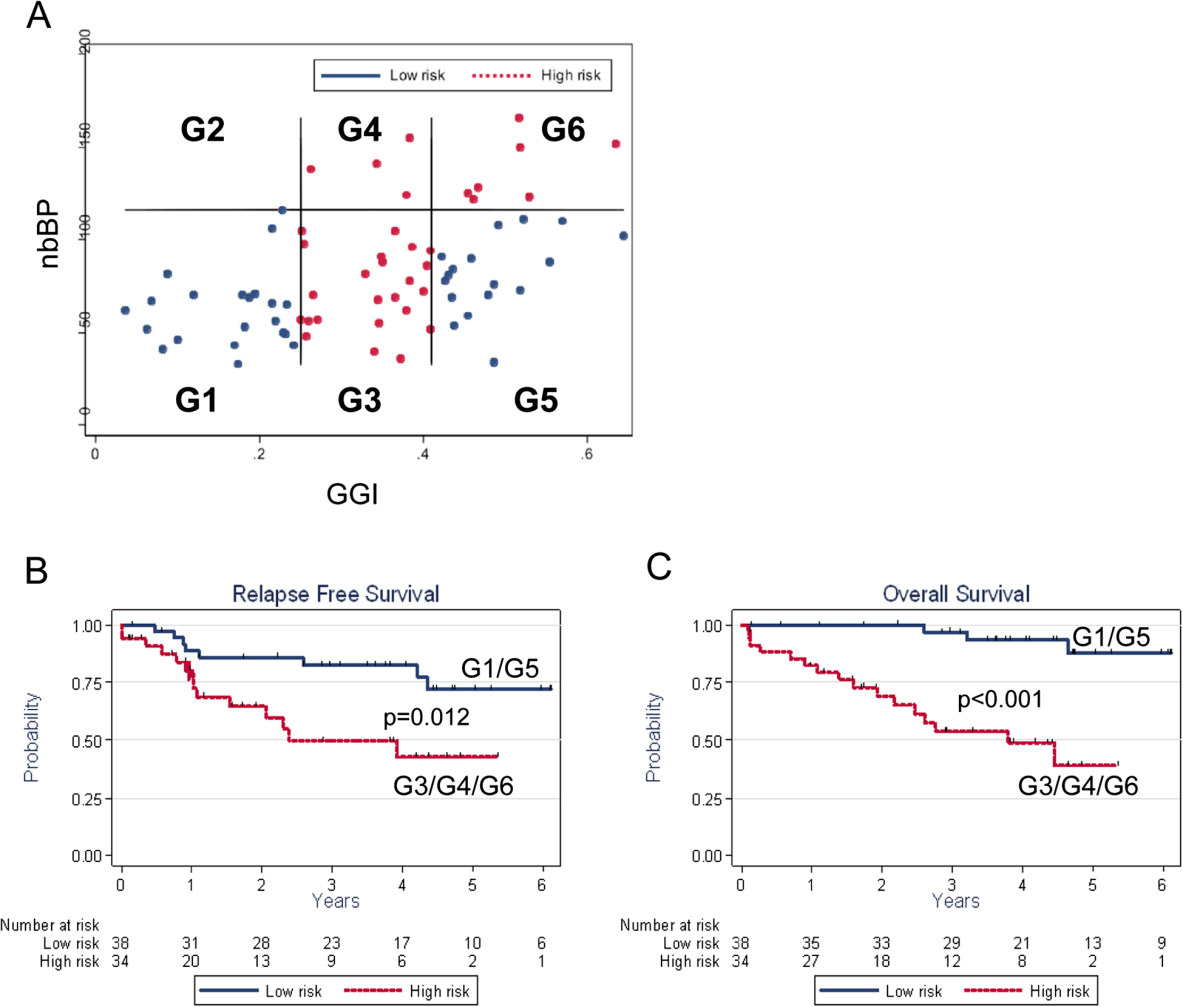
**Genomic instability risk groups in stage 2 and 3 CRCs.** Death and risk of relapse were correlated to genomic instability variables. **A**: Scatter plot integrating nbBP and GGI metrics. Three classes were determined for GGI:low (<0.25), intermediate ([0.25; 0.41[) and high (≥0.41) levels, with high risk associated to intermediate GGI, whereas two classes were defined for nbBP (<116 vs ≥116), with high risk being associated to high number of breakpoints. This produced 6 groups of risk of which one was empty. Tumors within high risk groups are shown as red dots, low risk as blue dots. **B**: Relapse free survival according to risk groups. **C**: Overall survival according to risk groups. Red curves correspond to the high risk, blue curves to low risk group.

While different stages of CRC could not be clearly distinguished by their cumulative CNA profiles, it was noticeable that individual tumors showed important differences in genetic instability, some tumors presenting highly rearranged genomes and others only limited numbers of anomalies. This prompted us to determine whether the level of genetic instability could be related to disease outcome. Because stage 1 tumors are associated to a very limited number of recurrence and stage 4 to negative outcome we focalized our analysis on the 72 stage 2 and 3 CRCs present in our dataset (17.5% stage 2 and 40.5% stage 3 CRC patients will eventually show disease progression at 5 years) [14] (see Additional file 7). Using ROC curves, we determined threshold levels for nbBP that were best fitted to define a group of bad outcome. This defined 2 classes, nbBP-low (<116) and nbBP-high (> = 116). The latter was associated to bad outcome in both RFS (p = 0.02) and OS (p = 0.001) (see Additional file 8A, B). For GGI, three classes were defined (low, medium, high) according to terciles. Best prognosis was found with GGI-low (<0.25) and worst prognosis with GGI-median [0.25-0.41]. Unexpectedly, GGI-high (≥0.41) tumors presented an intermediate outcome (RFS: <0.25 vs > =0.41, p = 0.3287; [0.25, 0.41] vs > =0.41, p = 0.1530; <0.25 vs [0.25, 0.41], p = 0.0220; OS: <0.25 vs > =0.41, p = 0.3843 ; [0.25, 0.41] vs > = 0.41, p = 0.0074 ; < 0.25 vs [0.25, 0.41], p = 0.002), see Additional file 8C, D). A multivariate Cox proportional hazards model was built based on the combination of GGI and nbBP. This model produced 6 groups (G1 to G6), of which only 5 were useful (G2 was empty) (Figure 3A). Using weights of the regression coefficients, we delimited 3 groups of high risk (G3, G4, G6) and 2 groups of low risk (G1 and G5). We combined all high risk and all low risk groups in one high and one low risk class, which showed clear differences in outcome (RFS: p = 0.012, OS: p < 0.001 Figure 3B, C) and Hazard Ratio using either relapse free (HR = 3) or overall survival (HR = 9.7) as an endpoint (Table 2).

**Table 2:** Survival rates according to prognostic categories

**RFS**				
	**No. of relapse**	**3-year RFS rate (%)**	**HR**	**95% CI**
Low risk	8/37	82.6	1	
High risk	15/35	49.8	3.0	[1.2;7.2]
**OS**				
	**No. of death**	**5-year OS rate (%)**	**HR**	**95% CI**
Low risk	3/37	87.9	1	
High risk	16/35	39.1	9.7	[2.8;34.0]

*Abbreviations*: *RFS* relapse-free survival, *OS* overall survival, *HR* hazard ratio.

*CI* confidence interval.

### CNAs associated to bad outcome in stage 2 and stage 3 CRCs

The above described risk classes were based on quantitative criteria (GGI and nbBP) defining levels of genetic instability in CRC. The different subgroups that were defined thus presented different levels of genetic instability (see Additional file 9A). However, while G4 (nbBP-High/GGI-median) and G5 (nbBP-Low/GGI-High) respectively belonged to the high and low risk class, their average numbers of gains, high level gains and losses were similar (Additional file 9B,C). This contrasted with G6 which bore distinctly higher numbers of CNAs.

These results prompted us to search for qualitative differences that may explain the differences in risk of relapse and death. We, thus, searched for specific copy number changes between high and low risk classes of CRCs, aiming at the definition of markers of relapse in stage 2 and 3 colorectal cancer.

**Figure 4:**
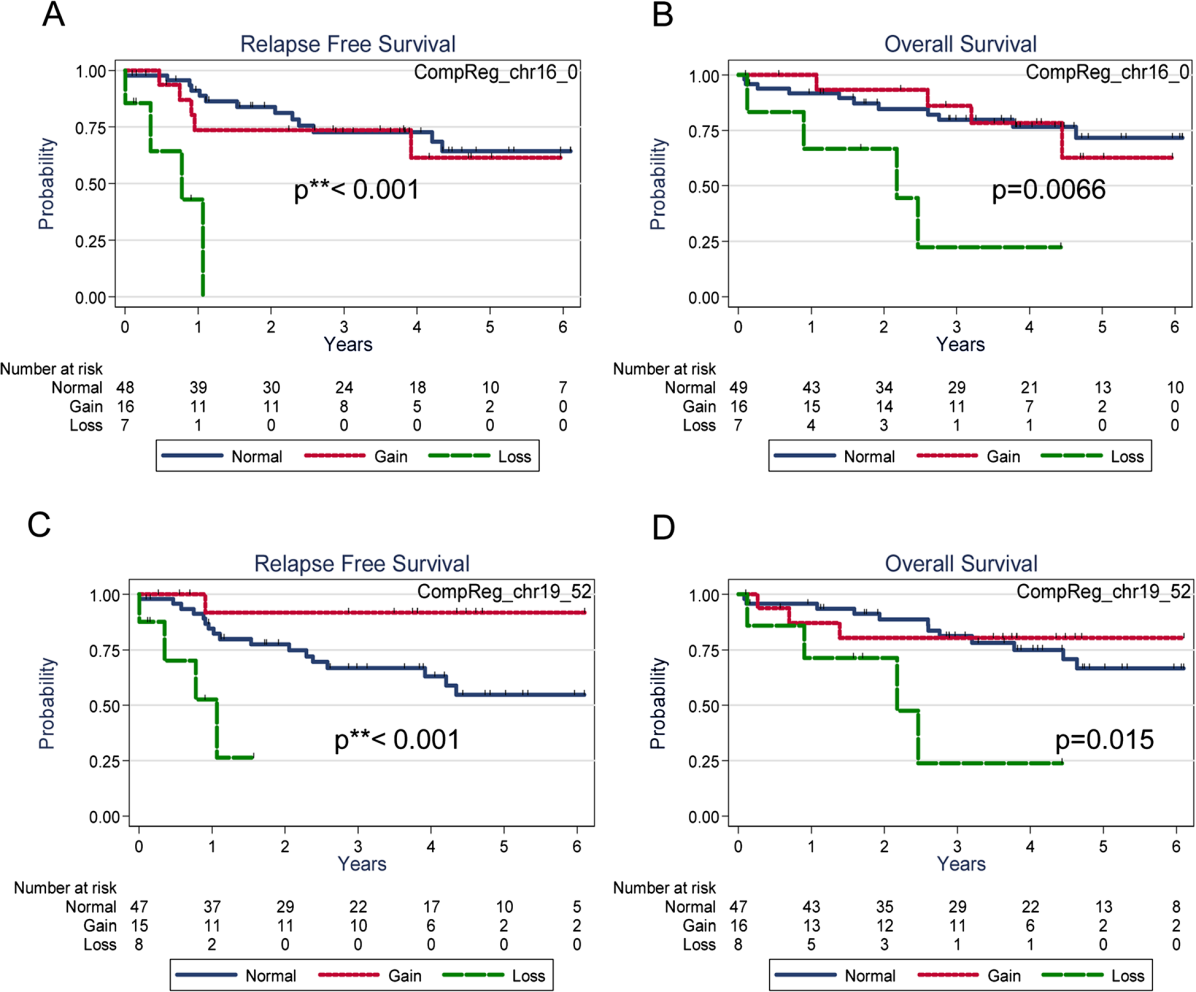
**Relapse free and overall survival according to CNA at 16p13.3 and 19q13.3 in stage 2 and 3 CRCs.** Losses at both locations are associated to shortened disease free and overall survival. Interestingly, gains at 19q13.3 appear to be protective for RFS. **A**: Relapse free survival according to CNA at 16p13.3. **B**: Overall survival according to CNA at 16p13.3. **C**: Relapse free survival according to CNA at 19q13.3. **D**: Overall survival according to CNA at 19q13.3. Red curves correspond to gain, green curves correspond to loss, and blue curves correspond to absence of CNA.

We used two complementary approaches. First we searched for significant differences in the high and low risk classes. Using the comparison tool of Nexus 6.0 software package we compared chromosomal regions of the high (n = 34) and low risk (n = 38) classes using two-tailed Fisher’s Exact test and identified 29 differentially represented genomic regions (see Additional file 10). Second, we used the Survival Predictive Power tool of Nexus in order to determine gains and losses significantly correlated with poor survival in the subset of stage 2 and 3 primary CRCs producing a list of 31 genomic regions (see Additional file 10). Losses at 16p13.3 and 19q13.3 were selected in both approaches and presented the strongest correlation (p < 0.001) to RFS and OS (p = 0.007, p = 0.016 Table 3, Figure 4A-D). Losses at 16p13.3 and 19q13.3 showed significant co-occurrence. All samples with 16p13.3 loss showed concomitant 19q13.3 loss (p < 1×10-8), thus signing for a group of CRC with negative outcome (see Additional file 11). This correlation remained significant in a subset of stage 2 CRCs (not shown). It was noticeable that CNAs at 16p13.3 and 19q13.3 were not restricted to losses and included a sizeable fraction of gains. However, only losses were associated to negative outcome, whereas gains had either no impact on survival risk (16p13.3) or, on the contrary for 19q13.3, were associated to favorable outcome (Figure 4C). This yin-yang correlation led us to investigate whether losses at 19q13.3 were enriched in high risk groups, while gains were more frequent in low risk groups. Indeed, we found that losses at 19q13.3 were enriched in the high risk group 6 (p = 0.0035), whereas gains were prevalent in low risk group 5 (p = 0.0025) (see Additional file 12).

**Table 3:** Survival rates according to genomic regions

**Genomic regions**	**S2S3PT (n=72)**		**RFS**				**OS**			
			**No. of relapse**	**HR**	**95% CI**	**3-year RFS rate (%)**	**No. of death**	**HR**	**95% CI**	**5-year OS rate (%)**
chr16p13.3	Normal	49 (68.1%)	13	1		72.8	11	1		71.8
chr16:0-2,410,722	Gain	16 (22.2%)	5	1.21	[0.43;3.41]	73.7	4	1.05	[0.33;3.31]	62.7
167 genes	Loss	7 (9.7%)	4	10.9	[3.10;38.6]	0	4	5.33	[1.63;17.5]	22.2
				p*=0.007		p**< 0.001		p*=0.05		p**=0.007
chr19q13.32-q13.33	Normal	47 (66.2%)	17	1		66.8	12	1		66.7
chr19:52,114,272-56,657,958	Gain	16 (22.5%)	1	0.17	[0.02;1.30]	91.7	3	0.77	[0.22;2.75]	80.4
285 genes	Loss	8 (11.3%)	4	5.04	[1.55;16.4]	26.3	4	4.36	[1.33;14.2]	23.8
	Missing	1								
				p*=0.003		p**<0.001		p*=0.07		p**=0.016

*Abbreviations*: *RFS* relapse-free survival, *OS* overall survival, *HR* hazard ratio, *CI* confidence interval, *, Likelihood ratio test; **, log-rank test.

In a second time, we tested in our stage 2 and stage 3 dataset the prognostic significance of focal regions and genes that were previously described in the literature [8,15,16]. Gains and losses were determined for each region bearing these target genes and association with RFS and OS tested. Significant association with short survival was found for only 5/87 genes, namely *SMAD4* (p = 0.0045)*, CCDC68* (p = 0.0054)*, TCF4* (p = 0.0054)*, RAX* (p = 0.0047) located on chromosome 18q21.2-21.3 and *TSKS* (p = 0.005) located on chromosome 19q13.3. Previously proposed prognostic regions such as losses at 4p, 4q22-q35, 5q, 6q, 8p, 13q ,14q, 15q, 17p, and gains at 8q, 10q, 20q were not found significantly associated with either RFS or OS in our series of stage 2 and 3 tumors [8,15-18].

## Discussion

In this work, using array-CGH as an analytical approach, we aimed at determining whether genetic instability was related to progression of colorectal cancer and verify whether it could be used as a prognostic indicator. Colorectal cancer has served as a model of stepwise progression from normal epithelium, through benign growth, into malignant cells that eventually become invasive and acquire metastatic properties [5]. We were interested in determining the existence of a progression scheme between superficial carcinomas (stage 1), invasive carcinomas (stage 2 and 3) and metastatic carcinomas (stage 4) based on a gradual accumulation of genomic alterations, with alterations occurring specifically at each step of progression.

Main regions of gain and loss observed in this work were concordant with previously reported observations on CRC [8,13]. Our data showing a marked increase in the number of CNAs in the transition from adenomas to carcinomas are in concordance with previously reported array-CGH work on early and advanced colorectal adenomas [11]. Most significant changes between adenomas and stage 1 CRCs were the occurrence of gains at 8q, 13q, 20 and losses at 8p, 15p, 17p and 18q in keeping with published works [7,11,19]. However, in contrast to Diep and coworkers [7] who proposed that transition from Duke’s B to C stage was associated to increased occurrence of gain at 1q and that Duke’s C to D to that of gains at 20q and Xq and loss at 21q, we could not identify CNAs whose occurrence was assigned to the transition from one stage to the next. This was exemplified by loss at chromosomes 14 and 15 which were present in stage 2, absent in stage 3 and present again in stage 4. We found that the number of CNAs in metastases was higher than in primary tumors. Analyzing concomitantly 28 pairs of primary tumors and corresponding metastases, we detected small regions of gains on chromosome 1q, 6p21, 10p and 17q21 and loss at chromosome 8p12 that occurred more frequently in metastases than in primary tumors (see Additional file 5). However, we could not infer the existence of anomalies specifying metastatic invasion as previously proposed [20]. Along similar lines we could not determine differences in CNA profiles between *TP53* wild type and mutated tumors [8]. The absence of specific changes associated to the *TP53* status in our dataset may be related to sampling differences. As a matter of fact, our series was restricted to CIN CRCs, while that in the work by Sheffer and colleagues (2009) comprised MSI cases that show *TP53* mutations at a lower frequency and present fewer CNAs and at different locations than in CIN tumors [21].

We could not associate any qualitative change of CNA to disease progression, but the increase of genetic instability between stage 1 and stage 4 tumors and between primary CRCs and metastases suggested that the global level of genetic instability could be of clinical or prognostic significance in CIN colorectal cancer. We were interested to note that whereas genetic instability was lowest in stage 1 and highest in stage 4 CRC, its level in stage 2, genetic instability was in keeping with that in stage 4. Taken together, our data were not consistent with a model where CRCs progress gradually from stage 1 to stage 4, because of the resemblance of CNA profiles and genetic instability levels in stage 2 and stage 4 tumors. This similarity suggested that a large part of stage 4 may arise directly from most unstable stage 2 tumors. The difficulty to define a progression scheme with increasing numbers of CNAs according to disease stages may be related to the fact that CRC staging is a clinical progression scale which takes local and distal invasion into account and no tumor intrinsic characteristics.

We, hence, asked the question of whether the level of genetic instability could be a prognostic indicator in stage 2 and 3 CRCs. Using the fraction of the genome involved in CNA (GGI) and the number of breakpoints (nbBP) detected in the array-CGH profiles, we defined 5 groups of CRC. These groups differed in their level of genetic instability, but also their profile of anomalies. Indeed, tumors with low nbBP presented CNA involving large chromosomal regions (whole chromosomes or chromosomal arms), whereas those with elevated nbBP showed fractional gains or losses. The group with the lowest instability (low-GGI/low-nbBP) was expectedly associated to good prognosis, whereas those with high-nbBP correlated with increased risk of relapse or shortened overall survival in stage 2 and 3 CRCs. Our results suggest that genetic instability could be an interesting tumor specific prognostic variable in CIN colorectal cancer. Along similar lines, Poulogiannis and coauthors [17] defined 4 groups of instability in CRC, with low levels of instability associated with good outcome and high levels with bad prognosis. It is of note that this study was performed on a series comprising both MSI and MSS CRC and it is likely that their group of low instability was largely composed of MSI cases, which are of better prognosis than MSS. Other studies have proposed to relate genetic CNA patterns to outcome [22] or response to chemotherapy [13]. Remarkably, in our study the group presenting an elevated fraction of the genome involved in CNA, but low breakpoint numbers (high-GGI/low-nbBP), was the other group associated with low risk. This group was representative of tumors with large regions of CNA or whole chromosomal arm copy variations and contrasted in terms of prognosis with tumors with high-nbBP which were of bad prognosis. These results were reminiscent of observations by Janoueix-Lerosey et al., [23], who showed that in neuroblastoma tumors with whole chromosome CNA displayed good survival, while those with fractionated CNA presented a high risk of relapse.

These data led us to search for specific copy number changes correlated to the bad outcome groups. To this aim we used two convergent strategies to identify regions of CNA correlated to adverse outcome in our stage 2 and 3 series. Copy number loss at 19q13.3 and/or 16p13.3 was clearly associated to worsened disease course, as shown by strong correlation with either shortened RFS or OS in stage 2 and 3 tumors. The 16p13.3 region has already been described as a prognostic region in CRC [15]. This region bears a total of 167 known genes among which *AXIN1* appears a serious candidate as it had been shown to be mutated in colorectal cancers and wild-type axin 1 can induce apoptosis in colorectal cancer cells [24,25]. The 19q13.3 region comprises 285 genes and among them the *BAX* pro-apoptotic gene and the polymerase delta gene *POLD1*. Low expression of BAX protein in stage 3 colorectal cancers has been linked to shorter RFS and 5-FU-based treatment resistance [26]. *POLD1* deletion could be involved in impaired DNA replication generating breaks and high rate of mutations [27]. As 16p13.3 (*AXIN1*) and 19q13.3 (*BAX*) are frequently co-deleted in our high risk group, apoptotic pathway in these tumors could be severely impacted giving significant resistance to apoptosis and growth advantage in malignant cells of these tumors. It was of note that we could confirm the association with adverse outcome for only 5/87 genes whose gain or loss had been previously shown to be of prognostic significance. Similarly, a large number of prognostic gains or losses could not be confirmed in our dataset [8,15-18].

## Conclusions

CNA profiles in CIN CRC are not consistent with the conventional scheme stating a stepwise progression from stage 1 to stage 4.

The level and pattern of genetic instability has been found to correlate with disease outcome, as tumors with fractionated gains and losses were of worse prognosis than tumors showing low breakpoint levels.

We identified that recurrent loss at 16p13 and 19q13 were significantly associated to bad outcome in stage 2 and 3 CRCs. Both regions were co-occurring in the high risk genetic instability groups.

## Competing interests

The authors declare that they have no competing interests.

## Authors’ contributions

BO carried out analysis and interpretation of data, participated in coordination of the study and drafted the article. JS participated in the conception and design of the work and revised the article. CBM performed the statistical analysis and revised the article. LL participated in array-CGH acquisition of data. KG participated in acquisition of data. FB participated in the conception and design of the study performed histological analysis. BM participated in acquisition of data. FP participated in the conception and design of the work. IS participated in acquisition of data. IH participated in acquisition of data. VD participated in acquisition of data. RG participated in the conception and design of the study and participated in acquisition of data. CC participated in the conception and design of the work and revised the article. ML participated in the conception and design of the work. CT participated in the conception and design of the work and drafted the article. All authors read and approved the final manuscript.

## Pre-publication history

The pre-publication history for this paper can be accessed here:

http://www.biomedcentral.com/1471-2407/14/121/prepub

## Supplementary Material

Additional file 1 Detailed patients and samples characteristics. Click here for file

Additional file 2 **ROC curve and determination of Youden’s index for nbBP in stage2 and 3 CRCs.** The optimal nbBP threshold was calculated using ROC curves to maximize the Youden’s index which induces the best discrimination according to vital status. Click here for file

Additional file 3 Multivariate Cox Model and score establishment. Click here for file

Additional file 4 Gain and loss regions found in more than 35% of the tumors. Click here for file

Additional file 5 Regions of CNA differentially represented in CRC disease stages. Click here for file

Additional file 6 **GGI (A) and nbBP (B) tercile distribution in clinical stages of 131 primary CRCs.** The GGI or nbBP values distribution were cut in terciles and the fraction of CRCs belonging to this tercile was plotted in Y axis for each clinical stage. S1: stage 1, S2: stage 2, S3: stage 3, S4: stage 4. The first tercile was colored in yellow, the intermediate one in orange and the last one in red. Stage 2 and 4 presented a large proportion of GGI-high and/or nbBP-high tumors, while stage 1 showed a prevalence of low instability tumors. Click here for file

Additional file 7 **Overall survival curves for the TNM stages of primary CRCs (n=129).** Stage 1 (black curve), Stage 2 (red curve), Stage 3 (blue curve) and Stage 4 (green curve). Click here for file

Additional file 8 **Relapse free survival and overall survival in stage 2 and 3 CRCs (n=72) according to nbBP groups and GGI groups.** nbBP groups were calculated using ROC curves (A,B) (nbBP<116; nbBP>=116; RFS (p=0.02), OS (p=0.001)) and GGI groups were determined according to distribution terciles (C,D) (GGI<0.25; GGI [0.25-0.41]; GGI>=0.41) (RFS: <0.25 vs >=0.41, p=0.3287; [0.25, 0.41] vs >=0.41, p=0.1530; <0.25 vs [0.25, 0.41],p=0.0220; OS: <0.25 vs >=0.41, p=0.3843; [0.25, 0.41] vs >=0.41, p=0.0074; < 0.25 vs [0.25, 0.41], p=0.002). Click here for file

Additional file 9 **Genomic groups characterization in stage 2 and 3 CRCs (n=72).** Gains are shown in green and losses in red. Boundaries of chromosomes are indicated by white and blue vertical areas. A: Frequency plots of CNA along the genome for the 5 genomic groups (G1, G3, G4, G5, G6) as defined in Figure 3. The high risk groups are G3/G4/G6 and the low risk groups G1/G5. B: Average number of regions of gains (green bars) and regions of losses (red bars) according to genomic group. G6 bore distinctly higher numbers of CNAs. C: Average number of high level regions of gain (green bars) and homozygous copy loss (red bars) according to genomic groups. G3 did not show homozygous copy loss in our series and despite a moderate GGI, G4 presented a number of high level gains similar to G5 and G6. Click here for file

Additional file 10 **Regions of CNA significantly associated to negative outcome in stage 2 and 3 CRCs.** Two lists were obtained using complementary approaches: 1- Comparison tool giving significant chromosomal regions in high and low risk classes using two tailed Fisher’s Exact test (p-value <0.005). 2- Analysis using the Survival Predictive Power package in the Nexus 6.0 genomic analysis software (perm p-value< 0.05). Two regions of loss were selected in both analyses and are highlighted in the list. Click here for file

Additional file 11 **Whole genome CNA frequency plots of stage 2 and 3 (n=72) CRC samples according to 19q13.3 CNA (gain, loss or no copy number changes).** Gains are shown in green and losses in red. Boundaries of chromosomes are indicated by white and blue vertical areas. Chromosome 16 and 19 are delimited by a black rectangle showing co-occurrence of 19q13.3 and 16p13.3 losses. Click here for file

Additional file 12 19q13.3 gains occur preferentially in risk group G5, whereas 19q13.3 losses correlate with high risk group G6. Click here for file
