# Supplementary material for: Impact of chromosomal instability on colorectal cancer progression and outcome
Source: BMC Cancer. 2014 Feb 22;14:121. doi: 10.1186/1471-2407-14-121 (PMC4233623; doi:10.1186/1471-2407-14-121)

Orsetti et al.
Additional file 6

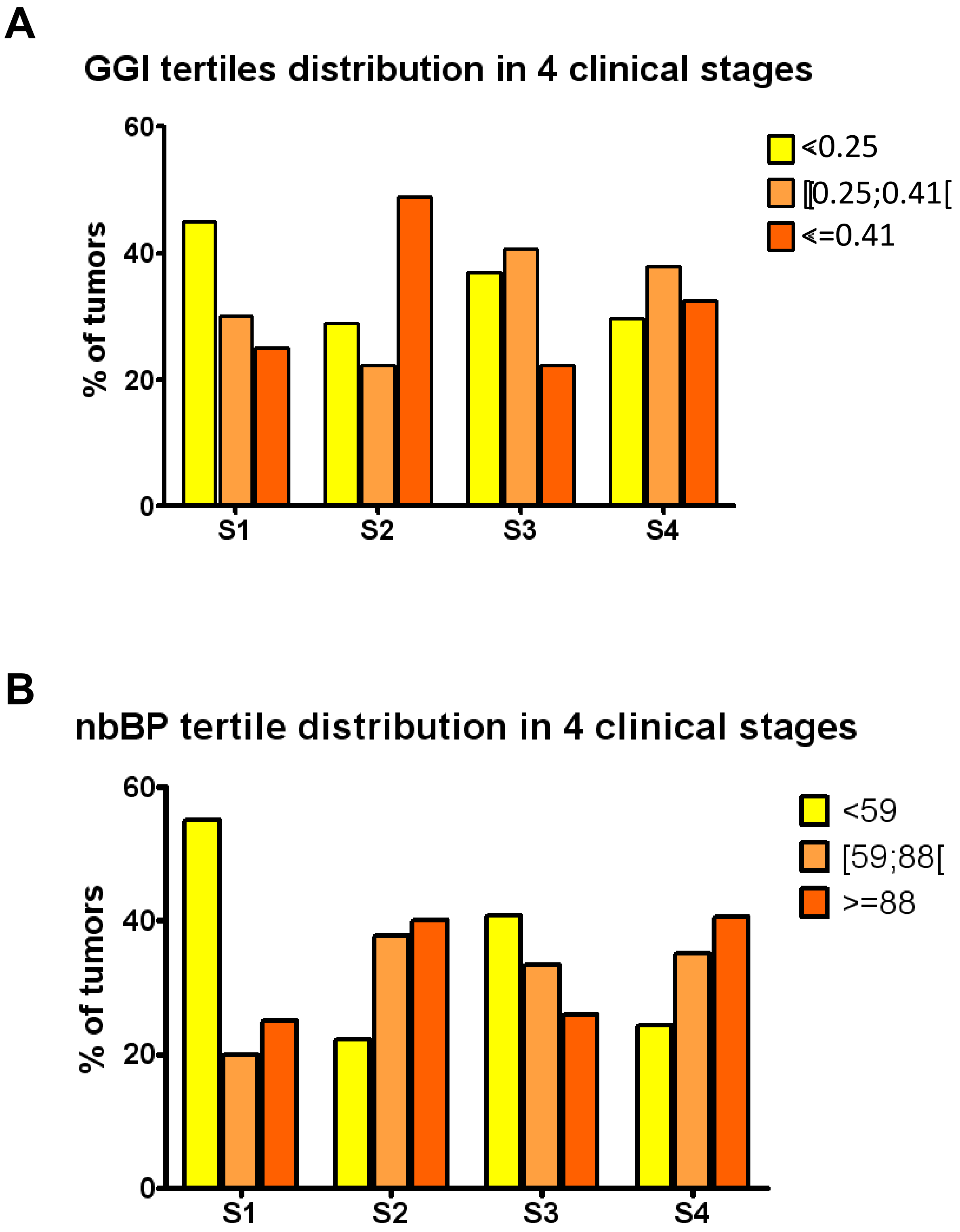

Supplement: Additional file 2 — ROC curve and determination of Youden’s index for nbBP in stage2 and 3 CRCs. The optimal nbBP threshold was calculated using ROC curves to maximize the Youden’s index which induces the best discrimination according to vital status. [file 1471-2407-14-121-S2.pdf]
