# Supplementary figures and images for: Impact of chromosomal instability on colorectal cancer progression and outcome
Source: BMC Cancer. 2014 Feb 22;14:121. doi: 10.1186/1471-2407-14-121 (PMC4233623; doi:10.1186/1471-2407-14-121)

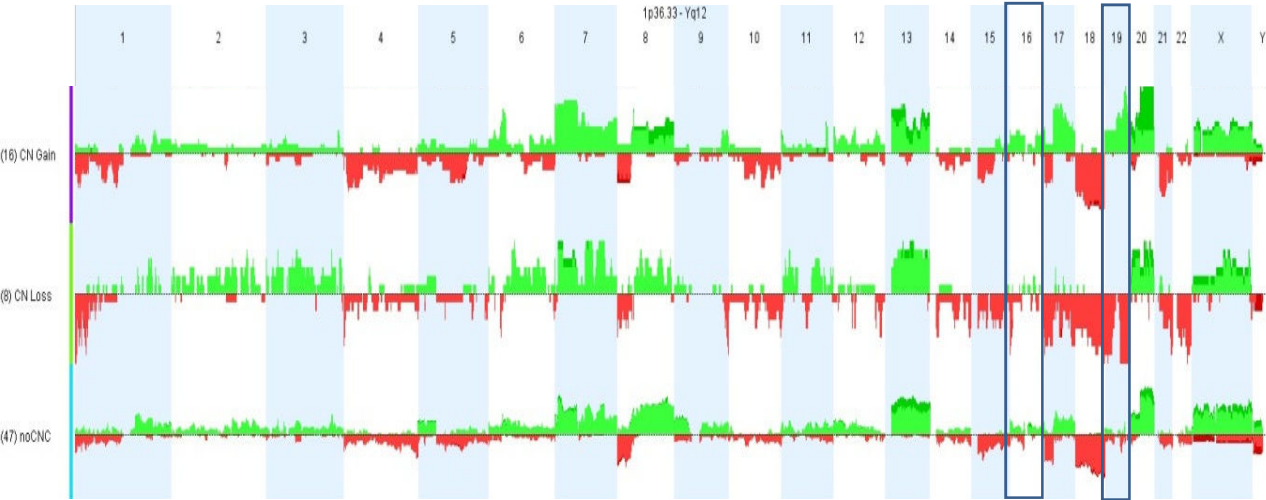

Supplement: Additional file 7 — Overall survival curves for the TNM stages of primary CRCs (n=129). Stage 1 (black curve), Stage 2 (red curve), Stage 3 (blue curve) and Stage 4 (green curve). [file 1471-2407-14-121-S7.pdf]

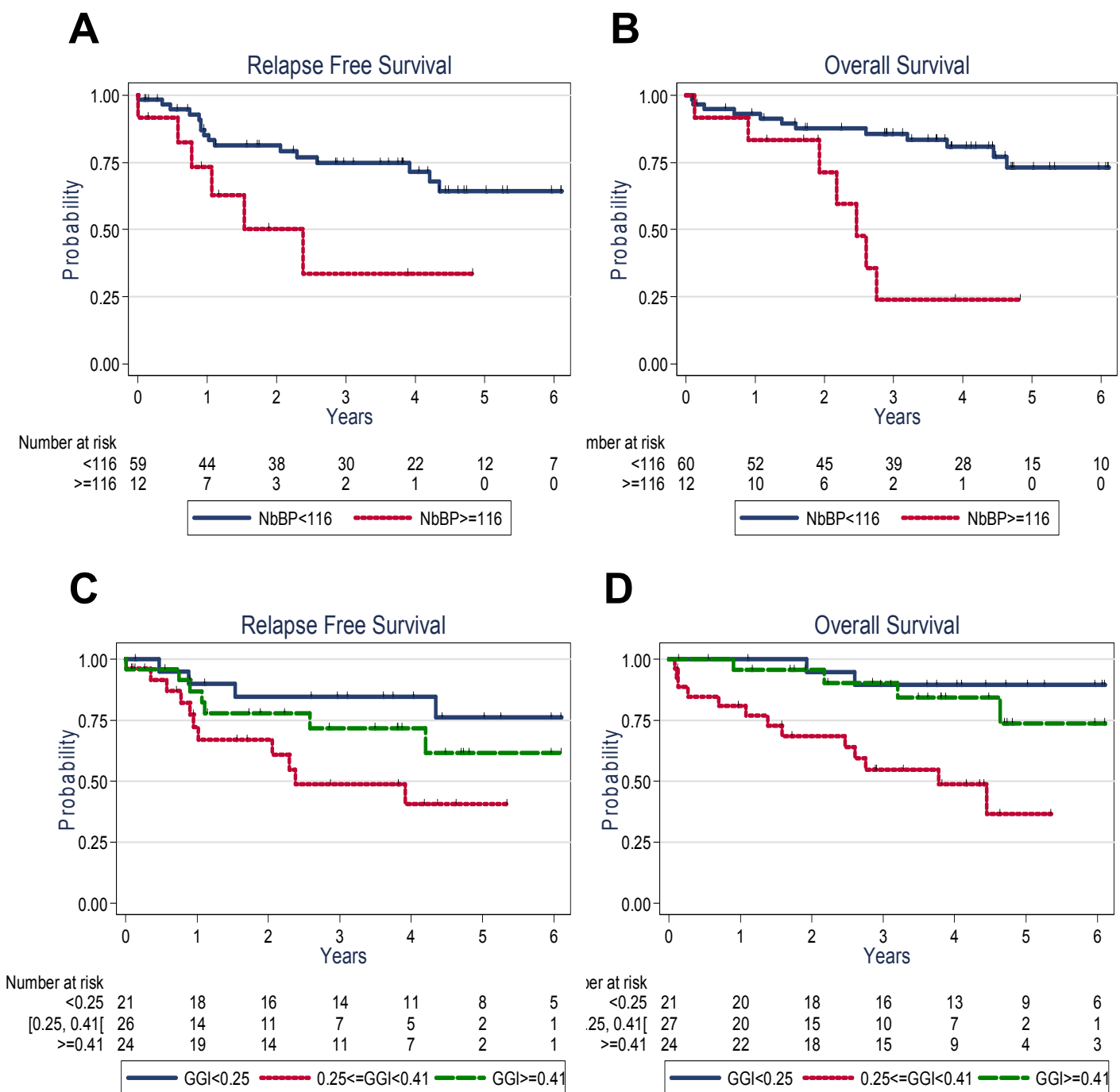

Supplement: Additional file 8 — Relapse free survival and overall survival in stage 2 and 3 CRCs (n=72) according to nbBP groups and GGI groups. nbBP groups were calculated using ROC curves (A,B) (nbBP<116; nbBP>=116; RFS (p=0.02), OS (p=0.001)) and GGI groups were determined according to distribution terciles (C,D) (GGI<0.25; GGI [0.25-0.41]; GGI>=0.41) (RFS: <0.25 vs >=0.41, p=0.3287; [0.25, 0.41] vs >=0.41, p=0.1530; <0.25 vs [0.25, 0.41],p=0.0220; OS: <0.25 vs >=0.41, p=0.3843; [0.25, 0.41] vs >=0.41, p=0.0074; < 0.25 vs [0.25, 0.41], p=0.002). [file 1471-2407-14-121-S8.pdf]

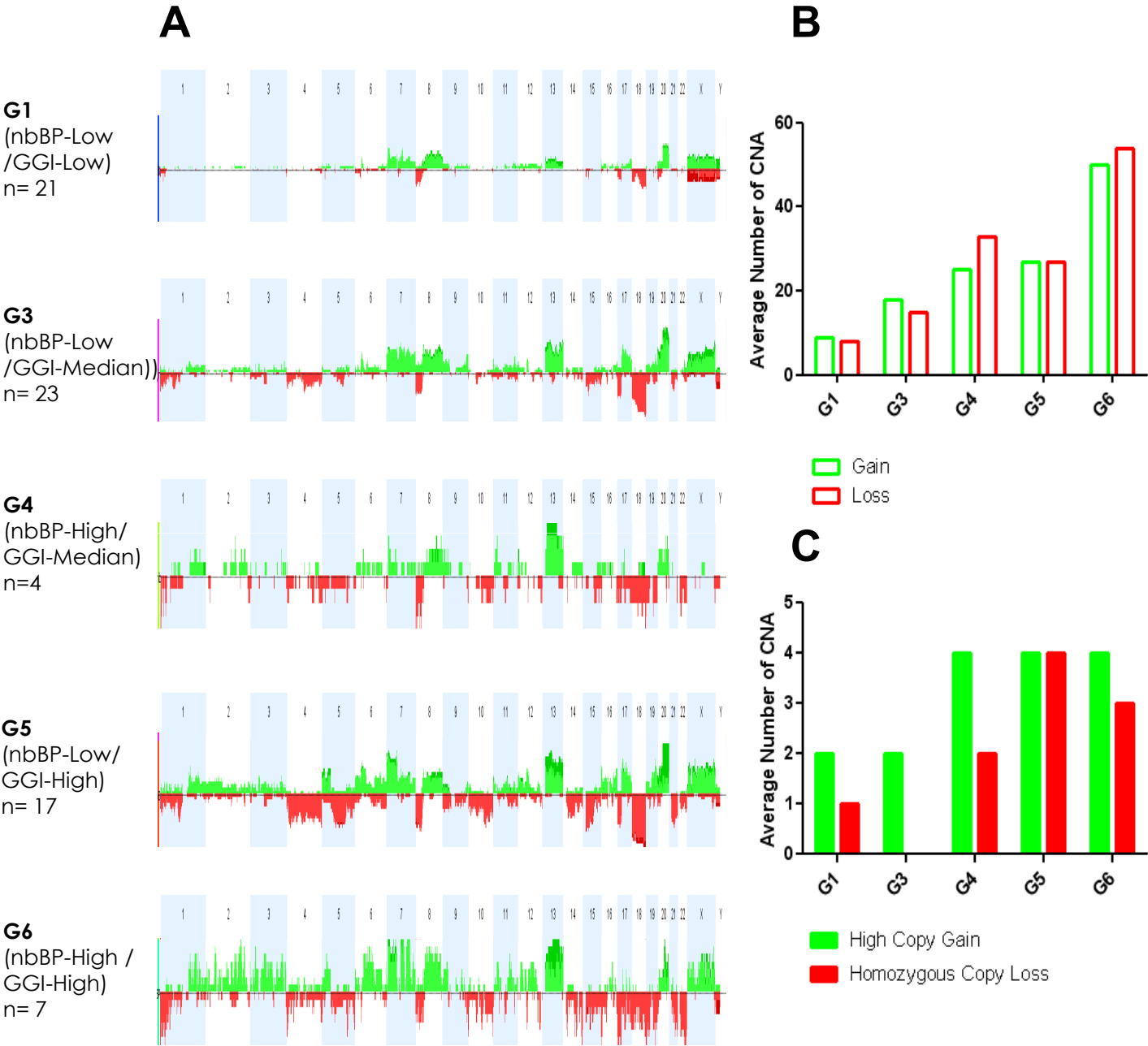

Supplement: Additional file 9 — Genomic groups characterization in stage 2 and 3 CRCs (n=72). Gains are shown in green and losses in red. Boundaries of chromosomes are indicated by white and blue vertical areas. A: Frequency plots of CNA along the genome for the 5 genomic groups (G1, G3, G4, G5, G6) as defined in Figure 3. The high risk groups are G3/G4/G6 and the low risk groups G1/G5. B: Average number of regions of gains (green bars) and regions of losses (red bars) according to genomic group. G6 bore distinctly higher numbers of CNAs. C: Average number of high level regions of gain (green bars) and homozygous copy loss (red bars) according to genomic groups. G3 did not show homozygous copy loss in our series and despite a moderate GGI, G4 presented a number of high level gains similar to G5 and G6. [file 1471-2407-14-121-S9.pdf]
